# FleXo: a flexible passive exoskeleton optimized for reducing lower back strain in manual handling tasks

**DOI:** 10.3389/frobt.2025.1687825

**Published:** 2025-10-23

**Authors:** Federico Allione, Maria Lazzaroni, Antonios E. Gkikakis, Christian Di Natali, Luigi Monica, Darwin G. Caldwell, Jesús Ortiz

**Affiliations:** 1 Department of Advanced Robotics, Istituto Italiano di Tecnologia, Genoa, Italy; 2 Department of Technological Innovation and Safety Equipment, Products and Anthropic Settlements, Italian Workers’ Compensation Authority (INAIL), Rome, Italy

**Keywords:** soft exoskeleton, flexible exoskeleton, wearable robotics, back-support exoskeleton, occupational exoskeleton

## Abstract

Musculoskeletal disorders, particularly low back pain, are some of the most common occupational health issues globally, causing significant personal suffering and economic burdens. Workers performing repetitive manual material handling tasks are especially at risk. FleXo, a lightweight (1.35 kg), flexible, ergonomic, and passive back-support exoskeleton is intended to reduce lower back strain during lifting tasks while allowing full freedom of movement for activities like walking, sitting, or side bending. FleXo’s design results from an advanced multi-objective design optimization approach that balances functionality and user comfort. In this work, validated through user feedback in a series of relevant repetitive tasks, it is demonstrated that FleXo can reduce the perceived physical effort during lifting tasks, enhance user satisfaction, improve employee wellbeing, promote workplace safety, decrease injuries, and lower the costs (both to society and companies) associated with lower back pain and injury.

## 1 Introduction

Musculoskeletal disorders (MSDs) are the most prevalent occupational diseases globally, with significant impacts on individual wellbeing and substantial economic burdens on healthcare systems and industries through increased medical costs, lost productivity, and compensation claims (Punnett and Wegman, 2004; Fatoye et al., 2023). Low back pain (LBP) is the most common MSD, ranking as the leading cause of disability worldwide in working-age groups (Hartvigsen et al., 2018), with 20–64 years being considered the working age by the European Union labor market statistics (Eurostat, 2025) and approximately 90% of the United States of America’s workers belonging to the same age group (U.S. Department of Labor, Women’s Bureau, 2024). Workers involved in repetitive and physically demanding manual material handling (MMH) tasks, such as repetitive lifting and carrying heavy loads, are particularly at risk of developing LBP (Coenen et al., 2014).

Back-support exoskeletons have emerged as effective tools to reduce lumbar loads and support users during strenuous MMH tasks (De Looze et al., 2016; Toxiri et al., 2019). They are not intended to improve the user capabilities but are designed to reduce physical effort during lifting by minimizing compression forces on the lower back, which can prevent injuries, reduce chronic LBP risk, and promote spinal health (OSHA, 2022; Marras et al., 1995; Norman et al., 1998). Proper lifting strategies alleviate strain on the spine, shoulders, and wrists, mitigating tissue overload and injury mechanisms linked to LBP. Additionally, reducing effort lowers the recurrence risk of injuries, with studies indicating that 44% of LBP patients experience a relapse within a year (OSHA, 2022).

Understanding force interactions with the human body is essential when designing exoskeletons. Industrial back-support exoskeletons use mechanisms categorized as rigid or soft. Rigid-frame exoskeletons, such as XoTrunk (Stadler et al., 2017), Laevo V2 (Van Harmelen et al., 2022), and GBS Apogee (German Bionic Systems GmbH, Augsburg, Germany), transmit forces perpendicularly to the spine, reducing vertebral compression. However, their concentration of reaction forces can create localized pressure on areas like the thighs and pelvis, reducing comfort (Kermavnar et al., 2021). They may also limit the user’s Range of Motion (RoM), restricting versatility.

Soft exoskeletons, including PLAD (Frost et al., 2009) and Apex (Lamers et al., 2017), try to improve comfort and user experience by spreading assistive forces over larger areas, but at the same time, they generate forces parallel to the spine, increasing the lower back compression of the vertebrae. Hybrid designs address some limitations of rigid and soft systems. For example, Spexor (Näf et al., 2018) uses lightweight, flexible carbon fibre frames to enhance RoM, while Yang et al. (2019) and Yang et al. (2022) developed a hyper-redundant hybrid cable-driven mechanism mimicking the human spine, enabling stoop lifting assistance without compromising mobility.

Exoskeletons are further classified into passive and active systems. Passive exoskeletons store and release energy through mechanical components such as springs or elastic bands (Abdoli-E et al., 2006; Näf et al., 2018; Alemi et al., 2019; Van Harmelen et al., 2022). While this is effective during lifting, they may reduce performance in tasks like walking (Baltrusch et al., 2019) by creating resistance to motion. In contrast, active exoskeletons employ powered actuators, such as electric motors or pneumatic systems, to provide tailored assistance (Aida et al., 2009; Lazzaroni et al., 2022; Yu et al., 2015; Inose et al., 2017). However, active systems are heavier (due to the mass of the actuators and possible onboard batteries), bulkier, and less robust due to reliance on external energy sources, limiting their usability in dynamic or external environments. Unlike active systems, passive exoskeletons avoid power source constraints, enabling extended practical use. This advantage drives the development of the FleXo exoskeleton. Its lightweight and ergonomic design makes it ideal for environments requiring mobility, comfort, and simplicity.

**FIGURE 1:**
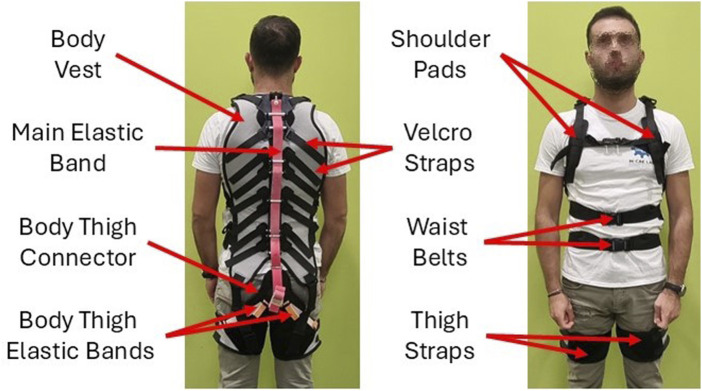
FleXo.

This work presents the design of FleXo, a lightweight, flexible, and passive back-support exoskeleton; see Figure 1. FleXo offers ergonomic back support for lifting tasks while preserving the user’s RoM for other activities, such as walking, sitting, twisting, or side bending, one of the main limitations of traditional passive exoskeletons. Its optimized design balances functionality and comfort by maximizing lifting support while minimizing vertebral compression. It is validated through user feedback during repetitive lifting tasks. FleXo aims to improve workplace safety, reduce injuries, and enhance wellbeing, mitigating the personal and economic burdens associated with LBP.

## 2 Materials and methods

This section outlines the methods used to design and validate FleXo. A multi-step process was employed, starting with user experiments to identify the optimal design for various tasks. A comprehensive design optimization approach then determined parameters that balanced comfort (minimizing injury risk) and performance (maximizing exoskeleton effectiveness). The final design was evaluated using standard questionnaires, and user feedback was analyzed to inform future improvements to FleXo.

### 2.1 FleXo design

**FIGURE 2:**
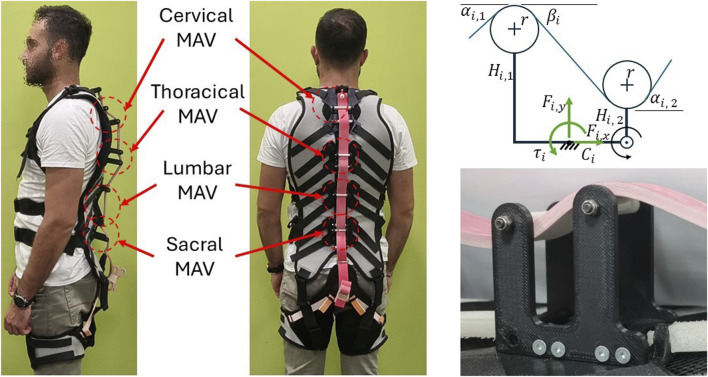
FleXo with 4 MAV modules (left). Schematic structure of a single MAV (right-top) and its physical realization (right-bottom).

FleXo is designed to support the user while lifting objects by reducing the overall effort without increasing the compression of the lower back vertebrae. To do so, FleXo’s mechanical structure is based on a chain of a patented mechanism called Modular Assistive Vertebra (MAV) (Ortiz et al., 2022; Allione et al., 2025; Fernández and Ortiz, 2020; Fernández and Ortiz, 2021). In each MAV, two pulleys are used to direct the transmission cable, made with an elastic band, into an ‘S’ pattern, as shown in Figure 2. This arrangement simplifies the mechanism by keeping it planar and ensures that the cable tension (T) generates a force parallel to the user’s spine $\left(F_{i,x}\right)$ , a force perpendicular to the spine $\left(F_{i,y}\right)$ , and a torque $\left(\tau_{i}\right)$ at the each MAV’s centre $\left(C_{i}\right)$ .

The human spine can be divided into five main sections: Cervical, Thoracic, Lumbar, Sacral and Coccyx. In the rest of this work, since all the vertebrae of the Sacral and Coccyx sections are fused, the two sections are considered as one and referred to as Sacral. Correspondingly, FleXo is designed with four independent MAVs named after each spinal section, as shown in Figure 2. Starting from the bottom, the first MAV is called Sacral, and it is located on the sacral section of the spine. The second MAV, called Lumbar, is located at the junction between the lumbar and the thoracic sections of the spine. The third MAV, called Thoracic, is located at the centre of the thoracic section. The fourth MAV, called Cervical, is located at the junction of the thoracic and cervical sections of the spine. Each MAV is rigidly mounted to a 3D-printed Acrylonitrile Butadiene Styrene (ABS) plate, which is secured to the garment using Velcro® straps.

Two consecutive MAV-plate structures are linked by a 3D-printed Thermoplastic Polyurethane (TPU) rod, which connects to a passive revolute joint on one side and is firmly attached to the other. The rod’s edges are cylindrical and inserted into cylindrical holes wide enough to allow for the rod to rotate, one into the rotational joint on one side and into the following MAV, allowing the MAV chain to adapt to the user’s twisting and side-bending movements while maintaining resistance to compression.

### 2.2 Problem formulation

**FIGURE 3:**
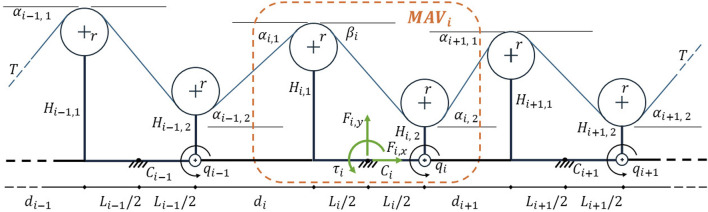
Schematic model of 3 MAV modules of FleXo (not to scale).

FleXo is a purpose-designed exoskeleton optimized to support the back while lifting objects. To maximize its performance, the geometry of each MAV is optimized in a multi-objective study. For each MAV, see Figure 3, the objectives are the following:

1. Minimize the compression on the user’s spine (Equation 1), and
2. Maximize the torque-to-force ratios (Equation 2).

This is mathematically described by the following equations:

$$\begin{array}{c} F_{\text{x}}/T=-cos\alpha_{1}+cos\alpha_{2}\;\\ F_{\text{y}}/T=-sin\alpha_{1}+sin\alpha_{2}, \end{array}$$ (1)

$$\begin{array}{c} \tau /T=\;+\left(sin\alpha_{1}+sin\alpha_{2}+2sin\beta \right)L/2\\ \;\;\;\;+\left(cos\alpha_{1}-cos\beta \right)\;\left(H_{1}+2r\right)\\ \;\;\;\;-\left(cos\alpha_{2}-cos\beta \right)\;H_{2}, \end{array}$$ (2)

where β is the angle between the elastic band and the pulleys of an MAV and is a geometrical property of each MAV. $\alpha_{1}$ and $\alpha_{2}$ are the angles between the elastic band and the pulleys of two consecutive MAVs and they are affected by the relative orientation of such MAVs. Both Equations 1 and 2 are derived and explained in detail in Allione et al. (2025). For each of the four MAVs, four parameters have been examined (see Figure 3):

• L the distance between the MAV’s pulleys,
• $H_{1}$ the height of the first (left) pulley,
• $H_{2}$ the height of the second (right) pulley, and
• d the distance between the pulleys of two consecutive MAVs (right pulley of ${\text{MAV}}_{i-1}$ and left pulley of ${\text{MAV}}_{i}$ ).

The radius r=3.5 mm of the pulleys, and the distance between the centers of two consecutive MAVs $\left(\bar{C_{i-1}C_{i}}=L_{i-1}/2+d_{i}+L_{i}/2=145\;mm\right)$ is given by the position of the Velcro® straps on the body vest, are defined from preliminary experiments not reported in this work. Since the elastic band is pushing the left pulley inward towards the spine and pulling outward the right one (see Figure 3), the constraint $H_{i,1}>H_{i,2}$ is added to the optimization algorithm.

For each MAV, the problem consists of 4 design parameters and 3 objectives, comprising 16 design parameters and 12 objectives for FleXo.

### 2.3 Sensitivity analysis

Sensitivity analysis identifies how variations in design parameters influence objectives, i.e., how much the design parameters of FleXo affect comfort and effectiveness, offering valuable insights to guide the design process. Although often overlooked, it helps uncover complex relationships in high-dimensional problems. In this case, the problem involves 16 design parameters and 12 objectives, governed by dynamics that vary significantly due to differences in anatomy, physical capabilities, load shape, and mass (Storey and Smith, 2012) of each user. For example, individuals employ distinct techniques for stooping or squatting. This analysis aids in pinpointing the most critical parameters, steering decisions toward optimal design choices.

For performing the sensitivity analysis, a Design of Experiments approach generated 20,736 uniformly-spread designs through a Full Factorial algorithm (Antony, 2023). Maintaining low correlation in the analysis design population is critical for accuracy, as the algorithm can detect correlated inputs (e.g., linear relationships among designs) and skew the results.

**FIGURE 4:**
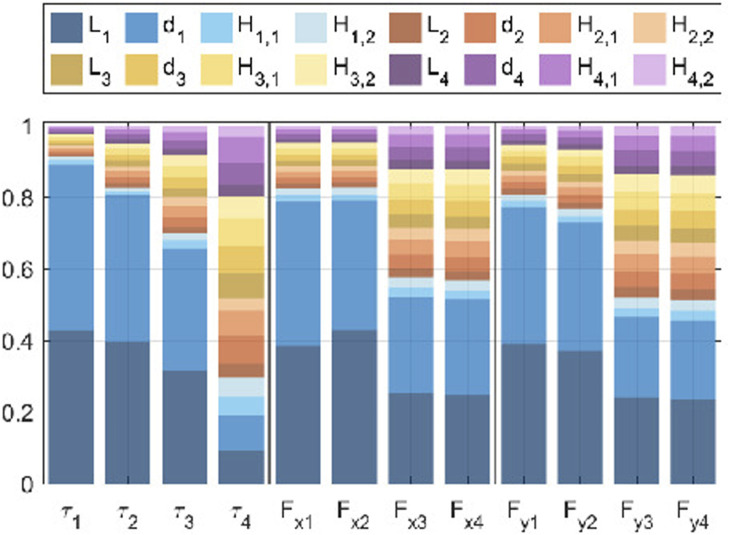
Colour scales refer to different MAVs: blue refers to the Sacral MAV, orange to the Lumbar MAV, yellow to the Thoracic MAV, and Purple to the Cervical MAV.

Figure 4 presents the results as a stacked bar chart, where each column corresponds to an optimization objective and the contributions of each parameter sum to one. Among the 12 objectives, the distance between the Sacral MAV pulleys $\left(L_{1}\right)$ and between the Sacral MAV and Lumbar MAV pulleys $\left(d_{1}\right)$ emerge as the most influential factors. These results are physically intuitive since these pulleys are closest to the hip’s rotation point. Notably, the influence of $L_{1}$ and $d_{1}$ diminishes with increasing distance from the hip, while the contributions of other parameters grow gradually. The remaining 10 parameters show close to equal and less significant effects. Consequently, the design of the first MAV is prioritized to minimize variations.

### 2.4 Garment

FleXo’s garment structure is crucial because it transmits forces from the exoskeleton to the user, see Figure 1. It is made of three different components: (1) a Body Vest, (2) a Body Thigh Connector, and (3) two symmetric Thigh Straps (one per thigh).

• *Body Vest*: The Body Vest is the interface between the user’s torso and the MAVs’ chain. Each MAV is rigidly connected to the Body Vest, which transmits the pulling force to the user via two shoulder pads and two waist belts. The main elastic band (elastic constant $K_{1}=521.2\;N/m$ , uniformly distributed throughout the applied elongation) passing through all the MAVs connects the Body Vest to the Body Thigh Connector. The vest is made of three layers: the first one is 3D air mesh fabric for breathability, the second one is made of Ethyl Vinyl Acetate (EVA) foam for structural rigidity, and the third one is Velcro®.
• *Body Thigh Connector*: The purpose of the Body Thigh Connector (BTC) is twofold. In the first place, it connects and transmits force from the Body Vest to the two Thigh Straps with elastic bands; second, it provides the user lifting force to the gluteal region when rising during a lift. It is located in the sacral zone, and it is made of three layers: the first two are the same as the Body Vest, while the third one is Nylon.
• *Thigh Straps*: The Thigh Straps serve as the anchor points where the tension force from the elastic cable, designed to assist with lifting, is transferred to the user’s legs. Connected to the BTC via two short elastic bands (elastic constant $K_{2}=362.5\;N/m$ , uniformly distributed throughout the applied elongation), these straps serve as fixed attachment points for the exoskeleton’s elastic components, allowing them to stretch and produce force during bending motions. The Thigh Straps are made of 3D air mesh fabric.

### 2.5 Design optimization

For the optimization study, experiments were designed and performed to identify each MAV’s trajectory while the user wears the exoskeleton while lifting an object. In the experiments, an operator wore the garment, with the MAVs being replaced by five Inertia Measurement Unit (IMU) (Movella DOT, Movella Inc., United States). Four IMUs measured the absolute orientation of the four MAVs, while the fifth was used to calculate the BTC’s orientation. The relative angle between each IMU has been derived (Allione et al., 2023), and the overall trajectory has been extracted and used for the optimization experiments.

The optimization methodology is based on the work of Gkikakis and Featherstone (2021), successfully applied to the design of legged robots (Allione et al., 2024; Allione et al., 2022). For optimization, Matlab’s *gamultiobj* (Coello et al., 2007) multi-objective evolutionary algorithm was used to explore the Pareto front, which is comprised of the designs with the optimal trade-off between comfort and effectiveness of FleXo. For initial designs, the middle value of the bounds was used, and the algorithm was allowed to execute for 100 iterations. Given that the main objective of FleXo is to reduce the compression on the user’s spine as much as possible while lifting weights, the optimization was more focused on minimizing the transmission of linear forces Fx among the multiple objectives. Given that the linear forces were already approximately one order of magnitude bigger than the torques, there was no need to use any weight or normalization factor. Table 1 reports the boundaries and optimal values found through optimization. The MAVs are 3D printed in ABS material, resulting in a total weight of FleXo of 1.35 kg , garment included.

**TABLE 1:** Optimization variables (Var) and the experiments’ lower (LB) and upper (UB) bounds.

MAV	Var	LB	UB	Optimal value
Cervical	$L_{4}$	20	100	51.0
	$H_{4,1}$	15	60	52.2
	$H_{4,2}$	15	60	25.1
	$d_{4}$	20	100	94.8
Thoracic	$L_{3}$	20	100	34.7
	$H_{3,1}$	15	60	56.7
	$H_{3,2}$	15	60	50.1
	$d_{3}$	20	100	96.1
Lumbar	$L_{2}$	20	100	98.5
	$H_{2,1}$	15	60	56.6
	$H_{2,2}$	15	60	31.4
	$d_{2}$	20	100	31.2
Sacral	$L_{1}$	20	100	45.3
	$H_{1,1}$	15	60	54.9
	$H_{1,2}$	15	60	24.5
	$d_{1}$	20	100	42.1

The Optimal Value column presents the results of the optimization study and the exoskeleton parameters used in this study. All values are in mm.

### 2.6 Experimental evaluation

As previously noted, current commercial systems, such as Laevo V2, can effectively provide back support while lifting; however, they limit the user RoM, limiting its ability to walk, sit or bend sideways freely. This experiment testing will seek to determine.

1. If FleXo provides useful support for users and has the potential to reduce LBP, and
2. How FleXo’s performance compares against commercial systems and specifically in these trials against the Laevo V2 system, which provides a ground comparison between the FleXo prototype and a commercially available exoskeleton.

### 2.7 Experimental protocol

The experimental analysis was conducted to compare and validate the feasibility of exoskeletons in assisting users in performing lifting and lowering tasks. Fifteen healthy men with no history of MSD were recruited (age: 28.7 ± 3.1 years, height: 182.6 ± 4.7 cm, weight: 77.6 ± 5.9 kg, Body Mass Index (BMI): 23,29 ± 2 kg/m^2^). To ensure unbiased results, none of the chosen participants contributed to the development of FleXo. The experimental procedure was approved by the Ethical Committee of Liguria (protocol reference number: CER Liguria 001/2019) and complies with the Helsinki Declaration. All the subjects received a full explanation of the experimental procedure and provided informed consent.

Participants were asked to complete lifting and lowering tasks using three techniques: Squat, Stoop, and Free, as shown in the accompanying video. The Squat technique is the most ergonomic and was defined as lifting with the knees flexed while maintaining the back as erect as possible. The Stoop technique is the least ergonomic and was described as lifting by bending the back and maintaining the knees as straight as possible. The Squat and Stoop techniques have a long history of research studies (Garg and Herrin, 1979; Straker, 2003; Arx et al., 2021); although the former is the generally suggested lifting technique, the latter is preferred by the subjects due to its lower metabolic cost (Van Dieën et al., 1999). However, while the squat is typically recommended as the safer and more effective method, it only results in lower net joint moments when the load is positioned between the feet. (Van Dieën et al., 1999). The Free technique consisted of using a self-selected technique that is usually an intermediate behavior between the two, in which both knees and back are flexed (Burgess-Limerick, 2003).

**FIGURE 5:**
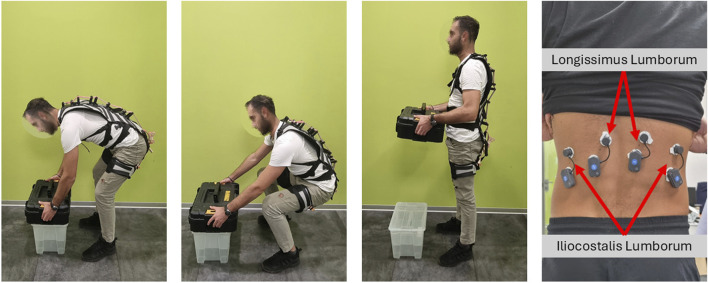
From left to right: stoop, squat, standing, and muscles instrumentally measured during a lift.

A single lifting and lowering task involved picking up a 10 kg box that was placed 40 cm above the ground, bringing it to an upright position, and then setting the box back on the support while returning to an upright stance. This process is illustrated in Figure 5. Each lifting and lowering task was repeated five times under every combination of technique and assistance conditions. The task cadence was set using a metronome, ensuring a rate of 10 lifting and lowering tasks per minute.

To ensure the safety of the subjects, the experimental procedure was designed according to the NIOSH safety requirements (Waters et al., 2021), where a Lifting Index (LI) below 1.0 indicates a safe activity, given by the following formula.

LI=L/RWL=10/12.37=0.81<1

where L = 10 kg is the Load Weight,

RWL=LC×HM×VM×DM×AM×FM×CM=12.37

is the Recommended Weight Limit, LC = 23 kg is the Load Constant, HM = 0.83 (30 cm horizontal distance of the weight) is the Horizontal Multiplier, VM = 0.9 (40 cm height of the handles) is the Vertical Multiplier, DM = 0.9 (55 cm vertical displacement of the weight) is the Distance Multiplier, AM = 1 (lifting trajectory entirely in the sagittal plane) is the Asymmetric Multiplier, FM = 0.8 (5 lifts per minute with work duration less than an hour and hands higher than 30 cm in starting position) is the Frequency Multiplier, and CM = 1 (optimal handle design) is the Coupling Multiplier, as described in Waters et al. (2021) § 1.3.

Subjects performed the task in four different assistance conditions:

1. Without the exoskeleton, referred to as *Noexo*;
2. Wearing the FleXo exoskeleton but with the elastic band not connected (i.e., not providing any support), referred to as *FNC*;
3. With the FleXo exoskeleton, referred to as *FleXo*;
4. With the commercially available Laevo V2 exoskeleton (Van Harmelen et al., 2022) with the assistance activated, referred to as *Laevo.*

The order of the assistance conditions was randomized over participants to reduce possible order-related confounding effects. Free lifting was executed first in each condition, while the Stoop and Squat order was randomized to limit the interference of the instructed lifts on the Free technique (Gill et al., 2007).

### 2.8 User assessment

To evaluate FleXo, participants were asked to complete two questionnaires. The NASA Task Load Index (NASA-TLX) questionnaire for assessing perceived workload during task execution and the QUEAD questionnaire for evaluating how well FleXo meets the user needs in terms of functionality, ease-of-use, safety, comfort, and overall satisfaction. To guarantee the reliability of the questionnaires, clear and consistent instructions for completing them were provided to all subjects, ensuring they correctly understood the questionnaires. Additionally, it was ensured that subjects were rested and undistracted while completing the questionnaires.

The unweighted NASA-TLX questionnaire was completed by participants after the end of each repetition for each combination of technique and assistance condition to assess the perceived workload. This questionnaire, developed by Hart and Staveland (1988), was demonstrated to be reliable for evaluating task workload (Hart and Staveland, 1988). It is more pragmatic and less sensitive to individual differences than the Subjective Workload Assessment Technique (SWAT) and more sensitive to workload differences than the Overall Workload survey (OW) (Grier, 2015). It utilizes six dimensions to evaluate the task workload: perceived mental demand, physical demand, temporal demand, performance, effort, and frustration. Each dimension is rated within a 20-point range. The NASA-TLX questionnaire, although it is not a standardized metric for evaluating occupational exoskeletons (Basla et al., 2022; Hussain et al., 2023), is increasingly being adopted as an alternative to custom-designed questionnaires or *ad hoc* surveys (Lazzaroni et al., 2023; Maurice et al., 2019), as, generally, standardized questionnaires are considered to produce more reliable results (Grazi et al., 2019).

The Questionnaire for the Evaluation of Physical Assistive Devices (QUEAD) was developed by Schmidtler et al. (2017) to assess physically assistive devices’ subjective overall acceptance and usability. The ISO standard defines usability as the extent to which specified users can achieve goals effectively and efficiently with satisfaction in a given context (ISO 9241-11:2018) (ISO, 2016). Usability is broken down into effectiveness, efficiency, and satisfaction. Effectiveness includes accuracy, completeness, and absence of negative consequences; efficiency is the relationship between results and resources used; and satisfaction encompasses positive attitudes, emotions during interaction, and comfort or discomfort from a physical perspective. It was crafted to assess perceived usefulness, ease of use, emotions, attitude, and comfort. Perceived usefulness and ease of use are critical for behavioral intention and acceptance of new technology (Davis et al., 1989). Comfort is particularly influential in the adoption of exoskeletons by end users (Hensel and Keil, 2019).

Finally, people’s assumptions about robots influence their satisfaction towards robots and intention to use (Ray et al., 2008; Nomura et al., 2005). Hence, in the questionnaire, users’ assumptions, in terms of attitude and emotions, are examined and measured. Its reliability, validity, and objectivity for perceived individual responses have been verified (Schmidtler et al., 2017). It ranks a total of 19 questions into five classes: Perceived Usefulness (PU), Perceived Ease of Use (PEU), Emotions (E), Attitude (A), and Comfort (C). All questions are assessed on a seven-point Likert scale from 1 (entirely disagree) to 7 (entirely agree).

### 2.9 Muscle activation

The assistance conditions were evaluated based on lumbar muscle activation and users’ kinematics, comparing results to the *Noexo* condition.

The lumbar extensor moment expresses the response of the musculoskeletal system to an external load applied and generates spine compression (Van Dieën and Kingma, 2005). Both peak and cumulative values of the extensor moment have been recognized as significant risk factors for MSDs during MMH activities (Marras et al., 1995; Norman et al., 1998). Research has demonstrated a clear association between lumbar extensor moment and the activity of the lumbar erector spinae muscles (Potvin et al., 1996; Dolan and Adams, 1993). As a result, Electromyography (EMG) recordings are widely employed to assess lumbar loading during MMH, particularly for tasks involving extended durations (Potvin et al., 1996). The erector spinae muscles are in fact the primary contributors to the extensor moment, although passive elements (such as intervertebral discs, spinal ligaments, the lumbo-dorsal fascia, and intramuscular collagen) also play a role in generating spinal compression forces (Dolan et al., 1994).

Surface EMG electrodes (BTS FREEEMG, BTS Bioengineering, Italy) measured the activity of the erector spinae (Iliocostalis Lumborum (IL) and Longissimus Lumborum (LL)) following SENIAM standards (Stegeman and Hermens, 2007), see Figure 5. EMG signals underwent band-pass filtering (10–400 Hz), with additional filtering to remove Electrocardiogram (ECG) artifacts (Drake and Callaghan, 2006) and electrical noise (notch filter at 50 Hz). Signals were low-pass filtered (2.5 Hz) and rectified to extract the envelope (Potvin et al., 1996). Data were normalized to the Maximum Voluntary Contraction (MVC) (McGill, 1991) for comparability across subjects and tasks. The MVC was determined by having participants perform a maximum exertion task three times: lifting their upper body against resistance while lying prone on a flat bench (McGill, 1991). Then, muscle activity averages were calculated using normalized data from the right and left sides.

Metrics computed included the Root Mean Square (RMS) and the 90th percentile during a single lift cycle, averaged for each subject and condition. RMS, representing signal power, indicates average muscle activity (De Luca, 1997), while the 90th percentile reflects maximum exertion during the cycle, providing robustness against outliers (Jonsson, 1982). RMS quantifies cumulative loading, linked to fatigue and musculoskeletal injury risks (Brereton and McGill, 1999), whereas the 90th percentile highlights peak loads, associated with acute intervertebral disc damage (Adams and Dolan, 2005). Together, these metrics comprehensively assess the exoskeleton’s impact on muscle activity and MSDs risk.

### 2.10 Kinematics analysis

A commercial Xsens-Awinda suit (Xsens Technologies, Enschede, Netherlands) recorded trunk and legs kinematics using eight wireless IMU attached to the trunk, upper and lower legs, and feet. The Xsens software reconstructed 3D biomechanical models and calculated joint kinematics. This analysis assessed the exoskeleton’s impact on RoM and natural movement patterns.

To investigate potential compensatory behaviors, the *FNC* condition was analyzed, where participants wore the FleXo exoskeleton without connecting the elastic element. This configuration tested whether factors like mass distribution or attachment fit altered natural movements or caused abnormal muscle activations.

### 2.11 Elastic band tension measurement

The tension on FleXo’s elastic band was measured during all the *FleXo* experiments with a Burster 8417-5500 series miniature tension and compression load cell (Burster Gmbh and co, Gernsbach, Germany). These measurements allowed us to estimate the forces and torques generated by FleXo, enabling a comprehensive evaluation of its mechanical performance.

### 2.12 Statistical assessment

Statistical analysis was conducted using JASP 0.18.3 (JASP Team, 2024), considering a confidence level of 95%. The one-way repeated measures analysis of variance (ANOVA) test was used to assess if there were statistically significant differences between the assistance conditions. When parametric assumptions were violated, the non-parametric Friedman’s test was used. Post hoc tests were performed with Bonferroni correction if the ANOVA or Friedman’s test was significant.

### 2.13 Ethics statement

The experimental campaign was carried out at XoLab (Wearable Robots, Exoskeletons and Exosuits Laboratory) at the Istituto Italiano di Tecnologia (IIT) in accordance with the Declaration of Helsinki; the experimental protocol was approved by the Ethics Committee of Liguria (reference number: CER Liguria 001/2019).

## 3 Results

### 3.1 Perceived results

The users’ subjective perception throughout the experimental procedure was assessed through the NASA-TLX questionnaires; the FleXo exoskeleton was also evaluated with the QUEAD, as detailed in the Materials and Methods Section. This analysis assessed perceived workload, usability, and overall user satisfaction.

### 3.2 NASA-TLX results

**FIGURE 6:**
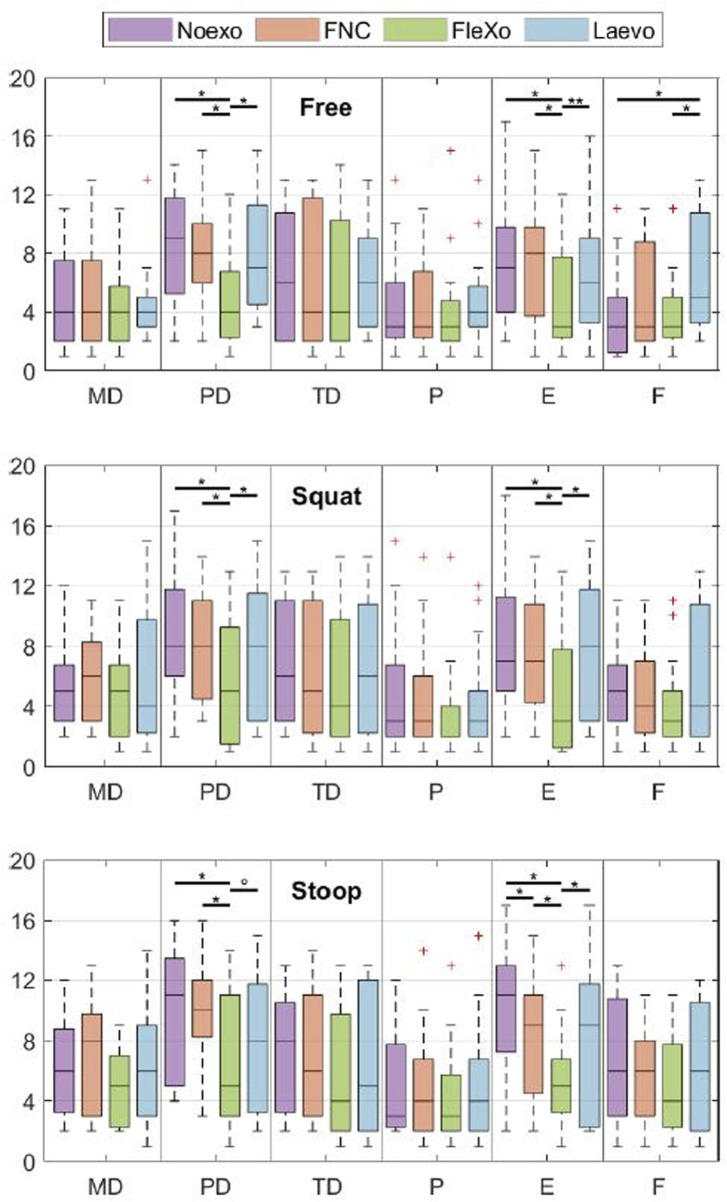
NASA-TLX questionnaire results expressed in a unitless 20-point range. MD is the Mental Demand, PD is the Physical Demand, TD is the Temporal Demand, P is the Performance, E is the Effort, and F is the Frustration. The median is shown as the central line, the box edges mark the 25th and 75th percentiles, and the whiskers represent non-outlier minima and maxima, with outliers as red crosses. Horizontal lines indicate the level of statistical significance where present.

Figure 6 presents the average scores for the three lifting techniques (Free, Squat, and Stoop). Due to violations of normal distribution assumptions, Friedman’s tests were used to assess the impact of assistance conditions on the questionnaire dimensions. Statistically significant differences were observed in the following dimensions.

• Physical Demand Significant reductions were observed across all lifting techniques when using *FleXo* compared to *Noexo* (p < 0.001), *FNC* (Free: p < 0.001; Squat: p = 0.031; Stoop: p < 0.001), and *Laevo* (Free: p < 0.001; Squat: p = 0.04; Stoop: p = 0.099).
• Effort Significantly decreased with *FleXo* across all lifting techniques compared to *Noexo* (Free: p = 0.008; Squat: p = 0.001; Stoop: p < 0.001). For Squat and Stoop, *FleXo* also outperformed *FNC* (Squat: p = 0.009; Stoop: p = 0.041) and *Laevo* (Squat: p = 0.006; Stoop: p = 0.019).
• Frustration In the Free lifting condition, *Laevo* induced significantly higher frustration compared to *Noexo* (p < 0.001) and *FleXo* (p = 0.021). Such a feeling comes from the constraints and reduction in the RoM induced by the Laevo V2 exoskeleton. FleXo, on the other hand, does not impose any limit on the users’ RoM, not inducing any frustration.

### 3.3 QUEAD results

Following the assisted lifting tasks with FleXo, participants completed the QUEAD questionnaire, with the results summarized in Table 2. Key findings include:

• Perceived Usefulness Participants rated FleXo highly in terms of enabling effective, efficient, and fast task performance.
• Ease of Use and Intuitiveness Users found FleXo easy to learn, use, and achieve desired outcomes with minimal effort.
• Comfort and Emotional Response The exoskeleton was not perceived as rigid, inflexible, or cumbersome. Positive evaluations of comfort, emotions, and attitude suggest a strong potential for user satisfaction in occupational settings.

**TABLE 2:** Results of the QUEAD questionnaire.

Perceived usefulness (PU)	
FleXo is useful	5.8 ± 0.7 [5, 7]
FleXo enhances my working performance	5.7 ± 0.9 [4, 7]
I accomplished the given task rapidly	5.7 ± 1.0 [4, 7]
I could efficiently complete the tasks using FleXo	6.3 ± 0.8 [5, 7]
I was able to perform precise motions with FleXo	6.1 ± 0.6 [5, 7]
I could effectively complete the tasks using FleXo	6.4 ± 0.6 [5, 7]
Perceived ease of use (PEU)	
FleXo is easy to use	6.4 ± 0.8 [4, 7]
It is easy to get the desired result with FleXo	5.9 ± 0.7 [5, 7]
FleXo is rigid and inflexible	2.2 ± 1.1 [1, 5]
FleXo feels cumbersome	1.9 ± 1.1 [1, 5]
I did not need concentration to use FleXo	4.8 ± 1.7 [2, 7]
I did not need physical strength to operate in FleXo	3.9 ± 1.5 [2, 6]
Using FleXo was intuitive	6.3 ± 0.6 [5, 7]
It was easy to learn to use FleXo	6.6 ± 0.5 [6, 7]
FleXo would be helpful to me	5.4 ± 1.2 [3, 7]
Emotions (E)	
I like using FleXo	5.2 ± 1.0 [3, 7]
I feel comfortable using FleXo	5.5 ± 0.8 [4, 7]
I feel unsettled by FleXo	2.1 ± 1.1 [1, 4]
I feel intimidated by FleXo	1.5 ± 0.7 [1, 3]
I feel anxious using FleXo	1.4 ± 0.7 [1, 3]
Attitude (A)	
I think that using FleXo is a good idea	5.6 ± 0.6 [5, 7]
I like collaborating in FleXo	5.8 ± 0.8 [5, 7]
I think I would use FleXo in future tasks	5.1 ± 1.1 [3, 7]
Comfort (C)	
I feel physically uncomfortable in using FleXo	3.2 ± 1.5 [1, 6]
I feel tense in using FleXo	2.7 ± 1.6 [1, 6]
I feel pain in using FleXo	1.2 ± 0.6 [1, 3]

Evaluations are on a scale from 1 to 7, where 1 is “Entirely Disagree” and 7 is “Entirely Agree” with the question. The questionnaire represents the users’ feedback for the combined Free, Squat, and Stoop experiments. Results are expressed in mean ± standard deviation; values in square brackets are the minimum and the maximum, respectively.

These results highlight FleXo’s ability to significantly reduce perceived physical workload and effort while maintaining high levels of user comfort and usability. The findings reinforce FleXo’s potential as an effective and user-friendly back-support exoskeleton for reducing MSDs risk during lifting tasks compared to Laevo V2, which proved to be not easy to use and cumbersome.

### 3.4 Instrumental results

Instructions were given to subjects to complete five lifting cycles for each combination of assistance and technique. The instrumental variables were measured during these cycles. However, the first and last lifts were excluded as incomplete or incorrect motions by subjects were often observed during those lifts. By considering only the three central lifting cycles, comparability among the repetitions was ensured.

**FIGURE 7:**
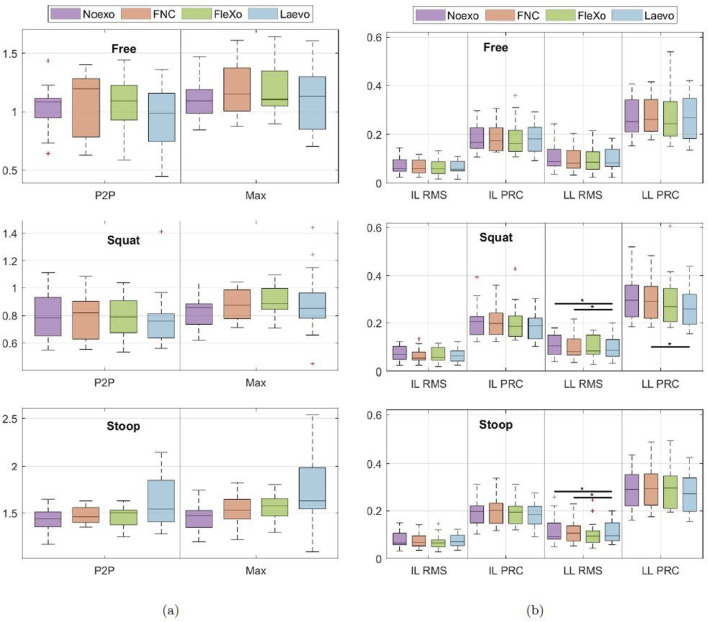
**(a)** Torso bending angle during a lift expressed in radians. P2P is the peak-to-peak torso bend angle (maximum bending minus vertical position), while Max is the maximum bending angle during a lift; values are in rad. No statistical significance is present. **(b)** Average value of the Root Mean Square (RMS) and 90th percentile (PRC) of the Ileocostalis Lumborum (IL) and Longissimus Lumborum (LL) activity during the three central lifts, normalized with the MVC,expressed as the percentage of the MVC. Horizontal lines indicate the level of statistical significance where present (p-value < 0.05).

The experimental procedure described above did not force the user to strictly follow a specific trajectory to avoid unnatural and annoying movements. As a result, the bending angle of the torso not only varied significantly among the subjects but also among the different exoskeleton configurations (*Noexo*, *FNC*, *FleXo*, and *Laevo*) and lifting techniques (Free, Squat, and Stoop), as shown in Figure 7a. However, the lack of statistical variation in bending angles among the various configurations highlights that the exoskeletons, hence FleXo, do not affect the user mobility during the lifting cycles.

Figure 7b shows lumbar muscle activity; more specifically, the normalized RMS and the 90th percentile of the muscle activity of the IL and LL during the three approaches to lifting-lowering, see Figure 5. The ease of the task and the relative freedom of motion allowed the subjects to have similar muscle activity of the back muscles, which were measured among all the configurations during the experiment. In the Squat experiment, subjects were instructed to keep their backs as straight as possible; hence, muscle activity did not show a statistically significant difference.

Despite that, subjects reported a perceived reduction in the general effort when using FleXo and, more specifically, for the leg muscles, which were not instrumentally measured. This is due to the presence of the BTC and the elastic bands connecting it to the Thigh Straps; when bending the legs, the elastic bands store energy that they release, helping the subject stand back up by pushing the BTC up.

**FIGURE 8:**
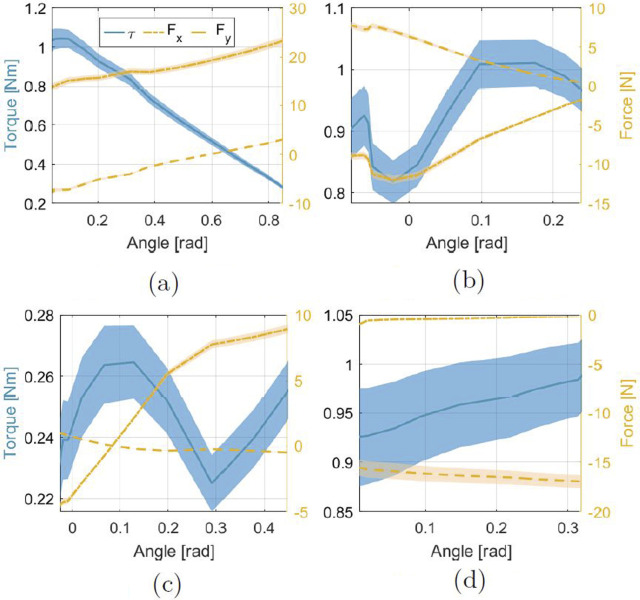
Estimated force and torque measurements of FleXo at each MAV for a Stoop bending, starting from a vertical position and going down to a maximum flexion. The angles represent the relative motion between two consecutive MAVs, while in the Sacral plot, the angle is defined between the Triangle and the Sacral MAV. **(a)** Sacral MAV. **(b)** Lumbar MAV. **(c)** Thoracic MAV. **(d)** Cervical MAV.

The force and torque profiles generated by each MAV can be estimated by combining the measured force of the elastic band during a Stoop experiment with the trajectory used for the optimization algorithm, as described in the Methods Section. These results are shown in Figure 8. It can be observed how FleXo can generate supporting torque while simultaneously limiting the compressing forces on the vertebrae. The compressing force generated by the Sacral MAV is around 5 N , equivalent to carrying an extra weight of approximately 0.5 kg while standing straight and a 2.4 N extending force while fully bent. The Cervical MAV is the only module that generates compressing force. This behaviour is intrinsic to FleXo’s structure, and it is because this particular MAV is the last in the chain, meaning that there is not a following MAV to compensate the horizontal component of the force as in Equation 1. Overall, these results prove the effectiveness of FleXo’s flexible design, showing its capabilities of generating lifting torque without increasing the compression on the vertebrae, similar to Laevo V2, which is based on a rigid structure.

## 4 Discussion

This work presented FleXo, a flexible, lightweight (1.35kg) passive back-support exoskeleton. Great attention was given to the user’s perception and feedback, and the results showed in the previous section validate FleXo’s design and implementation. Both the NASA-TLX and QUEAD questionnaires showed endorsing responses. The NASA-TLX highlighted a significant perceived reduction both in physical demand and effort to perform the lifting-lowering task when wearing FleXo without presenting any drawback or discomfort, including perceived lower back compression. Furthermore, the results are supported by the QUEAD that showed supportive feedback in all the categories. Moreover, FleXo did not cause noticeable impairments in the participants’ ability to bend during lifting activities, walk, sit, twist, or side-bend the torso.

Muscle activity reductions obtained with the Laevo V2 align with those obtained in a previous study when the device was tested using a similar experimental protocol—lifting and lowering a 10 kg box from mid-shin to upright (Koopman et al., 2020a). This study reduced peak back muscle activity by an average of −8% when using the Laevo compared to no exoskeleton. However, these reductions were not statistically significant across all conditions, as observed in our study.

Testing of the Spexor exoskeleton with a comparable experimental protocol reduced peak activity of the lumbar back muscles by up to 28% compared to no-exo (Koopman et al., 2020b). These greater reductions could be attributed to the exoskeleton’s large assistive torque (up to 50 Nm). However, the Spexor exoskeleton has other drawbacks: it is much heavier than passive exoskeletons (more than 6 kg). Furthermore, it significantly reduces the user’s RoM and trunk angular velocity when executing the tasks (Koopman et al., 2020b; Näf et al., 2018). Mean and peak EMG of the erector spinae muscles were significantly reduced by up to −16% and 23%, respectively, when using the Apex from HeroWear, an exosuit designed utilizing breathable elastic bands that weighs approximately 1.5 kg (Lamers et al., 2018). However, the tasks tested in this study have a higher level of physical demand than other experimental protocols: subjects were lifting two weights of 12.7 and 24 kg from the floor to a standing posture for 10 cycles.

Indeed, an exoskeleton’s benefits in reducing muscle activity may become more evident during extended usage and under tasks that involve higher physical demands (i.e., LI > 1). Future studies with FleXo are planned to investigate this scenario, which is likely to show statistically significant reductions in muscle activity. These findings align with previous reports indicating that increasing the load of lifted objects leads to more pronounced reductions in muscle activity (e.g. Lamers et al., 2018; Huysamen et al., 2018).

The physical size of the garment limited the audience of possible participants in the experiment. The location of the MAVs’ attachment to the body vest and the need to extend the elastic band sufficiently to generate a perceivable force required the user to be at least 1.75 m tall to use FleXo. Consequently, only male subjects were tested, given the difficulty of finding enough female subjects to meet such a requirement. The garment did not impose any limit to the maximum height or weight of the subject.

The current development stage of FleXo, coupled with the promising initial findings, suggests a potential for the device that necessitates further investigation in future work that will focus on improving both hardware and experimental procedures. On the one hand, a great focus will be placed on redesigning the Body Vest to accommodate a wider height range of users and transforming FleXo into a semi-active exoskeleton with dynamic stiffness adjustment based on user activity. Additionally, user feedback on perceived effort reduction will guide the measurement of leg muscle activation. Furthermore, conducting more realistic, task-oriented experiments will validate FleXo’s design more strongly. Finally, further studies on a more extensive and diverse group of people will be conducted to confirm the findings of this work.

## Data Availability

The raw data supporting the conclusions of this article will be made available by the authors, without undue reservation.
